# Natural dimethyl sulfide gradients would lead marine predators to higher prey biomass

**DOI:** 10.1038/s42003-021-01668-3

**Published:** 2021-02-01

**Authors:** Kylie Owen, Kentaro Saeki, Joseph D. Warren, Alessandro Bocconcelli, David N. Wiley, Shin-Ichi Ohira, Annette Bombosch, Kei Toda, Daniel P. Zitterbart

**Affiliations:** 1 Applied Ocean Physics and Engineering Department, Woods Hole Oceanographic Institution, Woods Hole, MA USA; 2 Institute for Marine and Antarctic Studies, Ecology & Biodiversity Centre, University of Tasmania, Battery Point, TAS Australia; 3 Department of Environmental Research and Monitoring, Swedish Museum of Natural History, Stockholm, Sweden; 4 Department of Chemistry, Kumamoto University, Kumamoto, Japan; 5 School of Marine and Atmospheric Sciences, Stony Brook University, Southampton, NY USA; 6 Stellwagen Bank National Marine Sanctuary, NOAA National Ocean Service, Scituate, MA USA; 7 Biophysics Lab, Friedrich-Alexander-Universtät Erlangen-Nürnberg, Erlangen, Germany; 8 International Research Organization for Advanced Science and Technology (IROAST), Kumamoto University, Kumamoto, Japan

**Keywords:** Behavioural ecology, Animal migration

## Abstract

Finding prey is essential to survival, with marine predators hypothesised to track chemicals such as dimethyl sulfide (DMS) while foraging. Many predators are attracted to artificially released DMS, and laboratory experiments have shown that zooplankton grazing on phytoplankton accelerates DMS release. However, whether natural DMS concentrations are useful for predators and correlated to areas of high prey biomass remains a fundamental knowledge gap. Here, we used concurrent hydroacoustic surveys and in situ DMS measurements to present evidence that zooplankton biomass is spatially correlated to natural DMS concentration in air and seawater. Using agent simulations, we also show that following gradients of DMS would lead zooplankton predators to areas of higher prey biomass than swimming randomly. Further understanding of the conditions and scales over which these gradients occur, and how they are used by predators, is essential to predicting the impact of future changes in the ocean on predator foraging success.

## Abstract (web summary)

Kylie Owen et al. sample concurrent prey biomass and natural dimethyl sulfide (DMS) concentration, and show that these variables are correlated in air and seawater. Agent simulations show that following fine-scale gradients of DMS would lead zooplankton predators to higher prey biomass, shedding light on how marine predators may use these cues for foraging.

## Introduction

Marine predators face the challenge of finding prey resources that are patchy and ephemeral in nature. Odour landscapes have been proposed as a strategy that animals may use to locate prey in a vast and largely featureless ocean^1,2^. The chemical compound that has received the most attention as a potential foraging cue is dimethyl sulfide (DMS). DMS in the ocean (DMS_aq_) and atmosphere (DMS_g_) are produced by the breakdown of dimethylsulfoniopropionate (DMSP), which is synthesised by phytoplankton^3,4^. DMS is the largest natural source of atmospheric sulfur and it plays an important role in cloud formation and likely in climate regulation^5^. Data collected in the context of climate science has shown that high concentrations of DMS occur in regions characterised by high productivity, being correlated to multiple factors such as chlorophyll-a concentration and mixed layer depth^6,7^. However, no previous research has determined whether natural DMS concentrations could accurately convey information on prey patch quality to a predator.

DMS concentration has been shown to be highly variable over both space and time^7^, with changes shown in response to meteorology, solar radiation, and phytoplankton photophysiology^8^. Many biotic (e.g. cell death, infections and phytoplankton species composition) and abiotic (e.g. temperature changes) factors are known to influence DMS production^9,10^. In addition, grazing by microzooplankton has also been shown to play a large role in DMS concentration in surface waters in some regions^11^. These species are too small to be prey for many large predators such as marine mammals, seabirds, and turtles that have been the centre of most behavioural research on the role of DMS in foraging. The high number of factors that influence DMS production, and large spatial and temporal variation in DMS concentration, may result in DMS being an uninformative foraging cue for predators. Despite all of the biological and oceanographic drivers of variability in DMS, for zooplankton predators to rely on DMS as an accurate foraging cue, an association between DMS concentration and prey biomass is essential.

Zooplankton grazing on phytoplankton has been shown to accelerate the rate of DMS release in laboratory experiments^12–14^. In addition, many marine predators, such as fish^15^, turtles^16^, marine mammals^17^ and seabirds^1,18^ have been shown to be attracted to artificially released DMS. This has led to the theory that zooplankton predators use this chemical to locate prey^1^ and the hypothesis that the attraction of predators to areas of DMS concentration may be the link between the sulfur, carbon and iron cycles in high latitude oceans^19^. However, the majority of studies on the behavioural response of predators to DMS have been completed in captivity or involved artificially introducing DMS into the environment^19^. To the best of our knowledge, the relationship between prey biomass and natural DMS concentration has never been compared in the field.

Here, we present concurrent high-resolution measurements of multiple phases of DMS (DMS_g_ and DMS_aq_) and prey biomass (zooplankton and fish), at a scale that is likely to be relevant to a foraging predator. Our aim was to determine whether there was a correlation between prey biomass and DMS and whether gradients of DMS existed at fine-scales, that could be utilised by predators to increase the prey biomass encountered while foraging. We found that zooplankton biomass and DMS concentrations in air and seawater are correlated and that smooth gradients of DMS exist at fine spatial scales that would lead zooplankton predators to areas of higher prey biomass in the ocean.

## Results

We sampled DMS (DMS_aq_ and DMS_g_) and prey (zooplankton and fish) biomass during surveys conducted during day time hours from Chatham Harbor, Cape Cod, MA, USA. These surveys covered a total of 220.2 km over 5 days in June 2019 (Supplementary Table 1, Supplementary Data 1, Fig. 1a–c). Over the 5 days, the DMS_g_ concentration ranged from 0.2 to 11.7 ppb (mean 4.4 ± 3.0 ppb (all means presented as mean ± SD)), and the DMS_aq_ concentration ranged from 1.3 to 19.5 nM (mean 8.0 ± 5.6 nM) (Supplementary Table 1). There were large differences in the concentration of DMS observed over consecutive days, and this was largely shadowed by the differences in the prey biomass encountered on the same day (Fig. 2a–d). Concentrations of DMS_g_ and DMS_aq_ were positively correlated (*n* = 193, *df* = 191, *r*_*s*_ = 0.80, *p* = 1.8e−44) (Fig. 3).

**Fig. 1:**
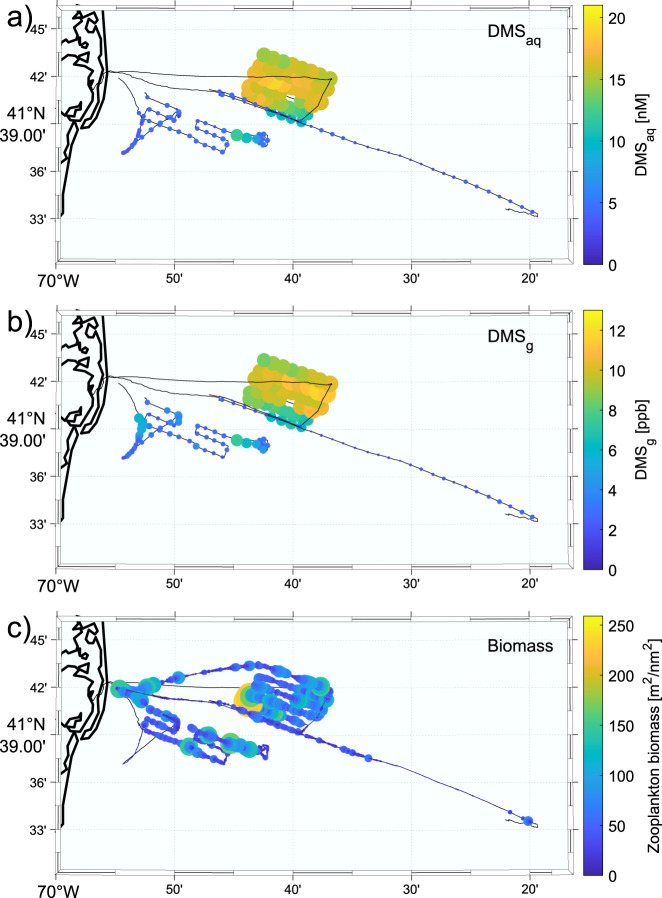
Dimethyl sulfide (DMS) concentration in seawater (DMS_aq_) and air (DMS_g_) along with concurrent acoustic measurements of zooplankton biomass (Nautical Area Scattering Coefficient (NASC)) off the coast of Cape Cod, MA, USA. The colour and size of the dots indicate the value of the measurement. Black lines represent the vessel track when no data were collected or where the measured value was zero. **a** DMS concentration in seawater (DMS_aq_). **b** DMS concentration in air (DMS_g_). **c** Zooplankton biomass (NASC).

**Fig. 2:**
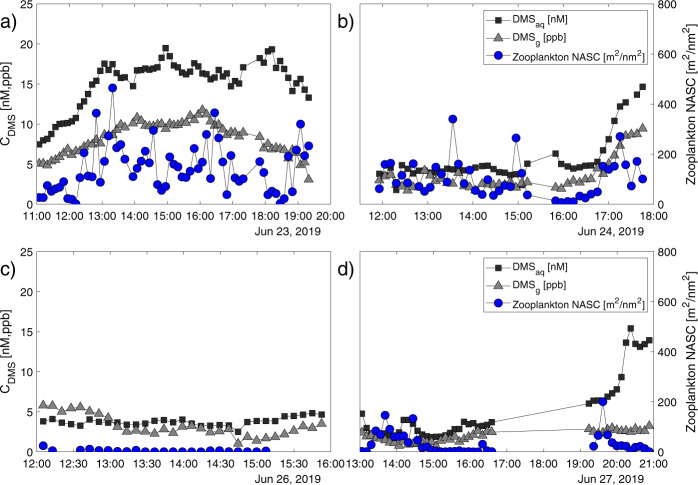
Dimethyl sulfide (DMS) concentrations in air and seawater and zooplankton biomass off the coast of Cape Cod, MA, USA, measured concurrently on four separate survey days. **a** A time series of all three measurements (DMS in air, DMS in seawater, and prey biomass) on 23 June 2019, **b** 24 June 2019, **c** 26 June 2019 and **d** 27 June 2019. There were large variations in all three parameters both within and among the days, although trends of high and low values for each parameter existed. Zooplankton biomass (as measured acoustically; Nautical Area Scattering Coefficient (NASC) (blue circles)) was more variable than either phase of DMS (DMS_aq_ in seawater (black squares); DMS_g_ in the air (grey triangles)) which may be the result of in situ natural variabilities. Only 4 days are shown as the duration of sampling was short on the fifth day (*n* = 14, ~1.5 h).

**Fig. 3:**
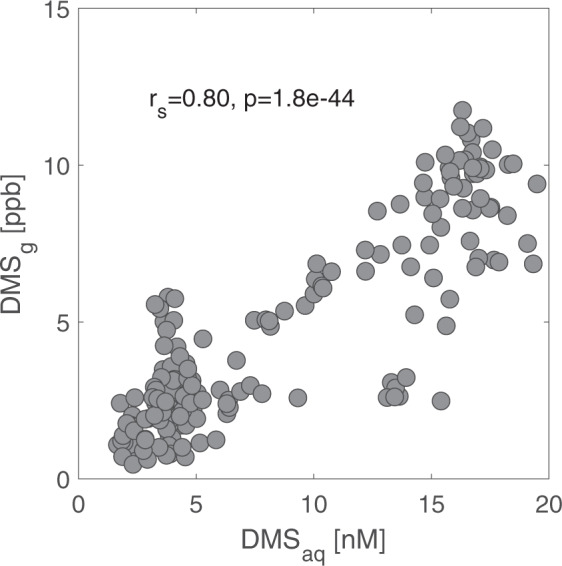
The correlation between two phases of dimethyl sulfide (DMS) in seawater (DMS_aq_) and air (DMS_g_). Measurements (*n* = 193) of both phases were highly correlated over 5 days of sampling off the coast of Cape Cod, MA, USA.

**Fig. 4:**
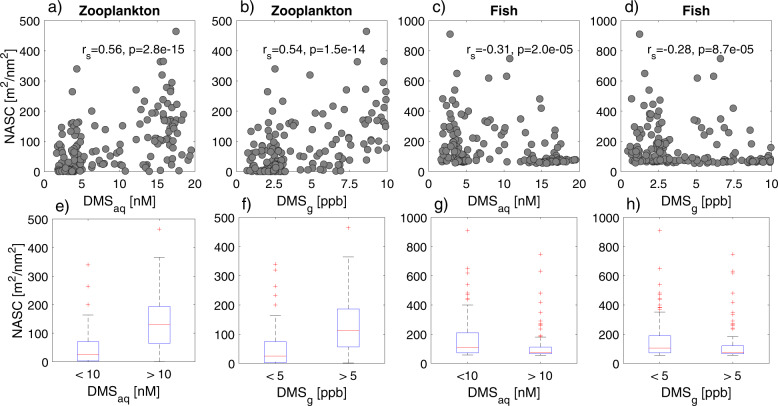
The correlation between dimethyl sulfide in the air (DMS_g_) and seawater (DMS_aq_) and acoustic estimates (Nautical Area Scattering Coefficient (NASC)) of zooplankton and fish off the coast of Cape Cod, MA, USA. The relationships varied between **a** zooplankton biomass and DMS in seawater, **b** zooplankton biomass and DMS in air, **c** fish biomass and DMS in seawater and **d** fish biomass and DMS in air. The panels (**e**–**h**) use the same data (*n* = 173 for **e**, **f** and *n* = 185 for **g**, **h**) as above and show the same relationships as the panels (**a**–**d**) but are split at the midpoint of the DMS range to highlight differences (or lack thereof) in zooplankton (or fish) biomass between low- and high-DMS values. Since zooplankton and fish NASC values are from different acoustic frequencies (710 and 38 kHz, respectively), the NASC values for the fish and zooplankton cannot be directly compared with each other.

Prey biomass (measured acoustically) ranged from 0.0 to 464.3 m^2^ nm^−2^ (mean 86.1 ± 90.0 m^2^ nm^−2^) for zooplankton and 55–910 m^2^ nm^−2^ (mean 153 ± 141 m^2^ nm^−2^) for fish. The concentration of both DMS_aq_ (*n* = 173, *df* = 171, *r*_*s*_ = 0.56, *p* = 2.8e−15) and DMS_g_ (*n* = 173, *df* = 171, *r*_*s*_ = 0.54, *p* = 1.5e−14) were found to be positively correlated to the biomass of zooplankton (Fig. 4a, b). Although this relationship is significant, the *r*_*s*_ is moderate, indicating that there is spread in the data. Areas of high zooplankton biomass did occur in regions with low DMS. However, when DMS was high (DMS_aq_ > 10 nM and DMS_g_ > 5 ppb; both values chosen at the midpoint of the range), there were rarely times when zooplankton biomass was low (close to zero) (Fig. 4e, f). In contrast, when DMS was low, zooplankton biomass was often low (Fig. 4e, f). The biomass of fish was weakly negatively correlated to both DMS_aq_ (*n* = 185, *df* = 183, *r*_*s*_ = −0.31, *p* = 2.0e−05) and DMS_g_ (*n* = 185, *df* = 183, *r*_*s*_ = −0.28, *p* = 8.7e−05) (Fig. 4c, d, g, h).

In order to test whether DMS gradients could be used by predators to locate areas of higher zooplankton biomass, despite the high spread in the data, we utilised agent simulations, where agents acted as pseudo-predators. There were two scenarios tested: (1) a tracking DMS experiment, where each agent was instructed to follow the DMS gradient, moving to the neighbouring cell with the highest DMS until a local maximum was reached, and (2) a random movement experiment, where agents were instructed to move randomly for the same length distribution as agents in the tracking DMS experiment. The distribution of the track lengths for the random movement experiment were limited to the same distribution as the tracking experiment for two reasons: (1) to ensure that the search effort was the same for both the random movement and tracking DMS experiments, and (2) as the maximum prey biomass encountered along each track was selected, having no limits on the length of the random movement tracks would allow all random agents to ultimately find the cell with the highest prey biomass in the entire area. The highest prey biomass encountered along all of the tracks was selected, as this is more likely to reflect true predator behaviour, with animals ending their search and beginning to feed as soon as prey is located. For the random tracking experiment, 1000 iterations were completed, and the mean of the maximum prey encountered across the 1000 tracks was compared to the tracking DMS experiment. Only one iteration was needed for the tracking DMS experiment as this is deterministic.

**Fig. 5:**
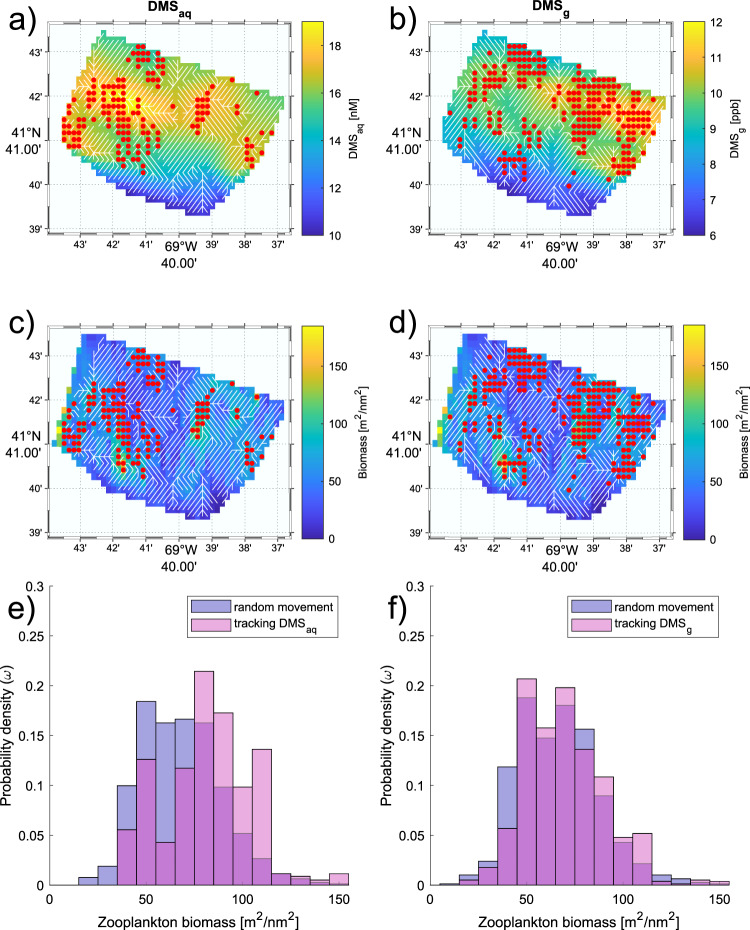
Agent simulation models of potential predator movement through gradients of dimethyl sulfide (DMS). Results are presented for seawater (DMS_aq_) and air (DMS_g_). Agent movements are shown as white lines, and the grid areas (*n* = 793) where the highest prey biomass was reached are shown as red dots. **a** Agent paths from the tracking DMS experiment in seawater overlaid onto DMS_aq_ concentration. **b** Agent paths from the tracking DMS experiment in air overlaid onto DMS_g_ concentration. **c** Agent paths from the tracking DMS experiment in seawater overlaid onto zooplankton biomass. **d** Agent paths from the tracking DMS experiment in air overlaid onto zooplankton biomass. **e** The distribution of prey biomass encountered while moving randomly (random movement experiment—mean of the maximum of 1000 iterations) (blue) and while tracking DMS_aq_ concentration (tracking DMS experiment- one iteration since it is deterministic) (purple). **f** The distribution of prey biomass encountered while moving randomly (random movement experiment—mean of the maximum of 1000 iterations) (blue) and while tracking DMS_g_ concentration (tracking DMS experiment—one iteration since it is deterministic) (purple).

Tracking DMS_aq_ concentrations lead agents to areas that had significantly higher prey biomass compared to when the agents moved randomly (DMS_aq_, 81 vs. 68 m^2^ nm^−2^, 19% higher, *n* = 793, *W* = 236747, *p* = 2.2e−16) (Fig. 5a, c, e). However, there was no significant increase in the maximum prey biomass encountered by agents tracking DMS_g_ compared to agents moving randomly (DMS_g_, 70 m^2^ nm^−2^ vs. 67 m^2^ nm^−2^, 4% higher, *n* = 793, *W* = 166,114, *p* = 0.1774) (Fig. 5b, d, f). When tracking DMS, the grid areas where the agents reached the highest prey biomass along their paths were largely congregated in areas of higher DMS concentration, and not necessarily in the grid areas with the highest prey biomass across the whole area (Fig. 5c, d). Across the same area, on average DMS_aq_ concentration led agents to significantly higher prey biomass than DMS_g_ concentration (81 m^2^ nm^−2^ vs. 70 m^2^ nm^−2^, 16% higher, *n* = 793, *W* = 41074, *p* = 2.2e−16).

## Discussion

We show that a correlation between zooplankton biomass and the concentration of DMS in air and seawater occurs in the ocean. Although it has been suggested that DMS is likely to be a useful foraging cue for marine predators, to the best of our knowledge, no previous research has demonstrated that higher concentrations of DMS are linked to higher prey biomass. The observed correlation between prey biomass and DMS is notable, especially given the many factors that influence the production and concentration of DMS^6,7,9,10^, and that DMS was sampled at a single depth while prey biomass was integrated over a larger depth range. Nonetheless, the occurrence of higher zooplankton biomass in regions of higher DMS suggests that natural concentrations of this chemical would allow DMS to act as a valuable foraging cue for zooplankton predators. Many marine predators, such as fish^20^, marine mammals^21^ and sea birds^22^ forage primarily on zooplankton, ranging from copepods to krill. Previous research has shown that attraction towards areas of DMS release is stronger in zooplankton predators compared to higher trophic level predators^23^. The lack of a positive correlation between fish biomass and DMS concentration may explain why higher trophic level predators show a weaker or no response to the chemical^23^. It is possible that time lags between DMS production and the build-up of fish biomass, or the diffusion of the chemical both spatially and temporally, may result in DMS being a valuable foraging cue only to zooplankton predators that will find higher prey biomass in areas of higher DMS concentration.

Higher prey biomass is likely to be a high source of DMS, which would then cause DMS to diffuse into the surrounding area and make the area of elevated DMS larger than the prey biomass area. We observed with the agent simulations that tracking gradients of DMS lead predators to areas that were characterised by higher DMS, but not necessarily the highest prey biomass in the area. Once in an area of high DMS and elevated prey biomass relative to the surrounding area, zooplankton predators may then initiate area-restricted search behaviour^24^, likely relying on a combination of senses to assist with finding the highest density prey patches in the localised area^24,25^. It is unlikely that DMS is used by predators in isolation, with predators using information from a number of sensory cues at different scales to reduce foraging effort. Examining how animals respond to the gradients of this chemical could provide much-needed information on the sensory capabilities of many marine predators. In addition, understanding the scales over which these gradients provide useful information on prey quality could also increase understanding of the scales at which different senses are used for foraging.

Based on optimal foraging theory, predators should use any method for finding prey that assists with reducing foraging costs, in order to improve foraging efficiency. It is thought that seabirds flying over the ocean are likely to rely on olfaction to smell DMS_g_ in the air as they forage over the ocean surface^26^. Here we have shown that tracking DMS_aq_ concentration would lead predators to areas of higher zooplankton biomass, but no significant benefit of tracking DMS_g_ was found. We also observed that DMS_aq_ lead agents to significantly higher prey biomass than DMS_g_, which was unexpected given the similarity in the strength of the correlation observed between prey biomass and both phases of DMS. We speculate that the difference in the success of DMS as a foraging cue in seawater vs. air is the result of the higher mobility of DMS_g_ in wind, relative to the movement of DMS_aq_ in seawater, making the spatial overlap between DMS_g_ and prey biomass less reliable. In addition, the data used for creating the DMS_(aq,g)_ maps took 8 h to collect, thus, especially for DMS_g_, it cannot be considered an instantaneous measurement. On the day of sampling, the wind direction was from west to east, which is evident in the eastward shift in the distribution of DMS_g_ relative to DMS_aq_ (Fig. 5). While flying at faster speeds than our survey vessel, seabirds may integrate or encounter regions of higher DMS_g_ differently than what our study measured. In addition, tracking gradients would likely result in a different spatial map than the transects completed in our study. If DMS_aq_ is more strongly associated with prey biomass than DMS_g_, tracking DMS_aq_ may be advantageous for larger animals, such as whales, that have energetically expensive feeding behaviours and require high prey biomass to feed efficiently^27^. Another possibility is that the use of DMS_g_ or DMS_aq_ may have different advantages for predators depending on the scale of the search. DMS_g_ is likely to provide information over broader scales than DMS_aq_, allowing for exploratory search behaviour over a broader area. However, further research is needed to determine whether DMS_aq_ consistently leads to higher zooplankton biomass than DMS_g_.

In this study, we focused on surface prey (in the upper 10 m) based on an assumption that prey in the upper water column would be most strongly associated with surface DMS_aq_ and DMS_g_ concentrations, and because zooplankton in the study area was predominantly distributed near the surface. Prey in this section of the water column is optimal for diving and air-breathing birds and mammals that are constrained by the energetic costs of diving^28^. As grazing by zooplankton accelerates the rate of DMS release^12,13^, it is also likely that the depth distribution of zooplankton influences the distribution of DMS concentration. Zooplankton species make diel migrations, coming to the surface at night to feed on phytoplankton, and moving to greater depths during the day to avoid predation^29^. DMS_aq_ concentration may therefore change diurnally in some areas and under certain conditions, yet, a universal diel pattern was not observed in the (sub-) tropical open oceans^30^. However, DMS_g_ concentration has been reported to be higher at night^31,32^, likely due to photochemical decomposition of DMS_g_ during the daytime. Our findings of a correlation between zooplankton biomass and DMS concentration suggest that variation in zooplankton grazing pressure in surface waters during the day and night may also influence DMS concentration throughout the day. Higher DMS concentrations at night could be a benefit to predators that may rely on vision to forage during the day and then switch to a stronger reliance on chemical cues in the dark. If animals can sense DMS_aq_, then the deeper exploratory dives completed by marine predators may be a mechanism to assist predators to locate zooplankton at depth. Understanding whether the exploratory diving behaviour of marine predators is also linked to DMS concentration may then provide insight into the energetics of marine predator foraging, particularly for air-breathing predators that may choose to continue or stop an exploratory dive based on the chemical information obtained during the dive.

We show that the link between DMS concentration and prey biomass that is necessary for predators to use DMS as an accurate foraging cue occurs in the ocean. This is a necessary condition to investigate which predators use these gradients to find prey and the scales over which DMS may be important to predators. How the use of these gradients interplays with other sensory modalities also needs further investigation, as it is likely that different senses are used at different scales^25^. It is worth noting that the results of this study were based on 5 days of field research in one location. Therefore, a priority for further research should be collecting additional data on concurrent DMS concentration and prey biomass in order to validate that the results are consistent in other conditions and locations. Much further research is required to fully understand the conditions under which these gradients occur, and the full range of oceanographic and biological factors that influence the strength of the correlation. For example, phytoplankton species composition is known to influence DMS production, and this factor may explain why in some areas prey biomass was high, while DMS concentration remained low. Alternatively, time lag effects could also play a role in explaining this pattern, with zooplankton (being longer-lived) biomass often remaining high long after phytoplankton has been consumed, and DMS has presumably dispersed. The variation in DMS concentrations observed in this study was relatively high despite the small observation area. This highlights the need for further fine-scale measurement of these spatially and temporally dynamic chemicals in order to fully understand their role in foraging ecology. Measuring the movement and distribution of marine predators in relation to natural DMS concentrations would provide some insight into whether predators respond to these gradients. Given the assumed link between DMS and global climate, understanding the role that DMS plays in predator foraging behaviour is essential to achieving an understanding of how the foraging success of predators may vary in changing oceans.

## Methods

### Study site

Data collection occurred over 5 days on board the *MV Noah* departing from Chatham Harbor, Cape Cod, MA, USA, during daylight hours from the 23 June to 28 June 2019. This is a known area of high primary productivity and a hotspot for baleen whale foraging activity^33^.

### Measurement of DMS concentration

Measurements of DMS in seawater (DMS_aq_) and air (DMS_g_) were conducted using a sequential vapour generation chemiluminescence instrument^34^. Water was collected from 1 m depth and approximately 1 m beside the vessel into an overflow tank with an exchange rate of 12 L min^−1^. We used a high flow-through pump and water in the overflow tank was replaced every couple of seconds. Air samples were collected via an inlet filter and a Teflon tube, attached to the roof of the pilothouse, well in front of the exhaust at the stern, in order to avoid contamination. Water samples were analysed for DMS_aq_ and gas samples were analysed for DMS_g_ automatically as distinct samples at a 7.5 min interval following a previously developed protocol^34^.

We conducted multiple transect protocols, including: (1) six transects in a lawn mowing pattern to obtain a 2D distribution of DMS concentration in order to conduct agent simulations, (2) targeting high and low productivity patches (based on echosounder data and observations of predators foraging in the area) in an attempt to cover a full range of possible prey biomass values in the area, (3) a drifting experiment using a sea anchor to stay in the same water mass and (4) a long-transect offshore and back. All data were used to assess the overall correlation between DMS and prey biomass.

### Measurement of prey biomass

Concurrent prey measurements were conducted using a multi-frequency down-looking echosounder (Simrad EK80 at 38 kHz and 200 kHz, both 18° beamwidth; Simrad ES60 at 710 kHz, 5° beamwidth). The EK80 was operated in narrowband mode and both the EK80 and ES60 echosounders operated with ping rates of 0.5–2.0 Hz (depending on bathymetry), pulse lengths of 256 microseconds, and output power of 500, 250 and 100 W (for the 38, 200 and 710 kHz systems, respectively) during the surveys. Echosounders were mounted on the port side of the vessel with the transducer faces at a depth of 0.5 m. All frequencies were calibrated during the study using a 38.1 mm diameter Tungsten carbide sphere^35^.

Because different organisms, such as small zooplankton and fish, scatter sound with different efficiencies depending on the acoustic frequency used, we used data from all three frequencies to measure the backscatter from small (<5 mm length) crustacean zooplankton such as copepods. In general, 38 kHz systems can detect fish with swim bladders, 200 kHz systems can detect fish without swim bladders and large mesozooplankton (such as krill > 20 mm), but neither can detect small copepods. However, small zooplankton produces measurable backscatter at 710 kHz^36,37^. To ensure that our acoustic measure of small zooplankton biomass was not including backscatter from individual or aggregations of fish, we identified regions of backscatter consistent with fish (volume backscattering strength > −70 dB) using the 38 and 200 kHz echograms, and then removed those same regions from the 710 kHz echogram before additional analysis. This procedure may lead to underestimates of zooplankton biomass as we may exclude some regions that had both copepods and fish present.

Because of this approach, we were limited in analysing data from regions where we had both 38 and 710 kHz data. The 38 kHz system has a larger nearfield region (roughly 2 m from the transducer) so we were limited to analysing backscatter data from 3 m and deeper. Since DMS measurements were made in the upper water column, we analysed acoustic data from 3 to 10 m water depths to try to best match the sampling region while also measuring sub-surface zooplankton aggregations. Backscatter at 710 kHz was binned (1 m vertically, 100 m horizontally) to roughly match the DMS sampling resolution and then vertically integrated (depths from 3 to 10 m) and converted to a zooplankton biomass value as measured by the Nautical Area Scattering Coefficient (NASC, m^2^ nm^−2^, a parameter widely used in fisheries acoustics as a proxy for zooplankton or fish biomass^38^), for each 8 m vertical by 100 m horizontal bin. Fish biomass was similarly estimated using the 38 kHz vertically integrated NASC data. We conducted three net tows using a 50 cm diameter, 2.5 m long ring net with 200-micron mesh towed 1–2 m below the surface for 2–5 min duration to collect ground-truth data on the fish and mesozooplankton present. Small copepods (lengths < 3 mm) were the most abundant taxa, although cladocerans, fish eggs and other small crustaceans were also caught. Pelagic fish aggregations were mostly mackerel which were caught by hook and line during breaks in the vessel survey operations.

### Statistics and reproducibility

The correlation between DMS and prey data was calculated based on the prey biomass bin that was collected concurrently to the DMS seawater and air sample (determined by the time of sampling). As the prey biomass data were not normally distributed, correlations were computed using a two-sided Spearman’s rank-order correlation within the corr function in Matlab (version R2019a). Significance was determined at *p* < 0.05 and exact *p* values are provided. The sample size for each separate correlation is provided in the “Results” section.

DMS and prey biomass maps (Supplementary Data 2) were computed using the grid data function with bilinear interpolation in Matlab. The 48.8 km^2^ area was sampled as a series of six transects and interpolated to a grid cell size of 0.0025°, equal to 208 m. Agent simulations were also computed in Matlab using the DMS maps and were used to represent pseudo-predator movement through the area^39^. We completed two experiments: (1) a tracking DMS experiment, and (2) a random movement experiment. As a starting point, one agent was placed in every grid cell (*n* = 783) across the whole area. In the tracking DMS experiment, each agent was instructed to move from their initial position to an adjacent grid cell with the highest DMS concentration (DMS_aq_ or DMS_g_) out of the eight grid cells adjacent to its current position. This behaviour was repeated until they reached a local maximum, where all of the adjacent grid cells had a lower DMS concentration than the current grid cell. We expect real predators would stop their search behaviour, with their movements no longer following the DMS signal, when prey is located, as opposed to the agents that continued searching until a local maximum in DMS was reached. As a result of this, the grid cell along the path that had the highest prey biomass was labelled and used to derive a frequency distribution of maximum prey biomass encountered when tracking DMS. In addition, a random movement experiment was completed, where an agent started in each grid cell but was instructed to move randomly. The length of the tracks for the random movement experiment was pulled from a distribution that was equal to the distribution of track lengths in the tracking DMS experiment. This was to prevent random agents from searching the entire area and always encountering the highest prey biomass in the area and to ensure that the search effort by agents was consistent in both experiments. The tracking DMS experiment was only run once, as it is deterministic, however, the random experiment was completed 1000 times from each grid cell. The maximum prey biomass encountered was recorded for each of the 1000 random tracks from each cell. The mean of the maximum prey encountered over these 1000 iterations was then compared to the maximum prey encountered in the tracking DMS experiment.

Since prey biomass measurements are likely to be spatially correlated and thus individual grid cells are not necessarily independent, and agents beginning in the same grid cell (random movement and tracking DMS) could be considered paired samples, non-parametric Wilcoxon signed-rank tests were completed in R Statistical Software (version 3.6.1) to compare the mean of the maximum prey biomass encountered by agents moving randomly, to the mean of the maximum prey biomass encountered when the agents followed either DMS_g_ and DMS_aq_ (*n* = 783). In addition, the same test was used to compare the mean of the maximum prey biomass encountered when the agents followed DMS_g_ to the mean of the maximum prey biomass encountered when following DMS_aq_.

## Supplementary information

Supplementary Information

Description of Additional Supplementary Files

Supplementary Data 1

Supplementary Data 2

## Data Availability

All data generated or analysed during this study are included in this published article (and its Supplementary Information files).

## Code availability

The code used to generate the findings of this study^39^ are available under DOI: 10.6084/m9.figshare.13286216.
