# Supplementary material for: Natural dimethyl sulfide gradients would lead marine predators to higher prey biomass
Source: Commun Biol. 2021 Feb 1;4:149. doi: 10.1038/s42003-021-01668-3 (PMC7851116; doi:10.1038/s42003-021-01668-3)
Supplement: Supplementary file 1 — Supplementary Information [file 42003_2021_1668_MOESM1_ESM.pdf]

Supplementary Information for:

Natural dimethyl sulfide gradients would lead marine predators to higher prey biomass

Kylie Owen[1,2,3], Kentaro Saeki[4], Joseph D. Warren[5], Alessandro Bocconcelli[1], Dave Wiley[6], Shin-Ichi Ohira[4], Annette Bombosch[1], Kei Toda[4]* and Daniel P. Zitterbart[1,7,8]*

[1]Applied Ocean Physics and Engineering, Woods Hole Oceanographic Institution, Woods Hole, MA, 02543, USA
[2]Institute for Marine and Antarctic Studies, Ecology & Biodiversity Centre, University of Tasmania, Battery Point, TAS, Australia, 7004
[3] Department of Environmental Research and Monitoring, Swedish Museum of Natural History, SE-104 05, Stockholm, Sweden
[4]Department of Chemistry, Kumamoto University, 2-39-1 Kurokami, Kumamoto 860–8555, Japan
[5]School of Marine and Atmospheric Sciences, Stony Brook University, 239 Montauk Hwy, Southampton, NY 11968 USA
[6]Stellwagen Bank National Marine Sanctuary, NOAA National Ocean Service, 175 Edward Foster Road, Scituate, MA 02066, USA
[7]Biophysics Lab, Friedrich-Alexander-Universtät Erlangen-Nürnberg, Henkestrasse 91, 91052 Erlangen
[8]International Research Organization for Advanced Science and Technology (IROAST), Kumamoto University, 2-39-1 Kurokami, Kumamoto 860–8555, Japan

*corresponding authors

Supplementary Table 1: Details of dimethyl sulfide (DMS) and prey biomass measurements taken off Cape Cod, USA over five days in June 2019. DMS was measured in both air (DMS_g) and seawater (DMS_{aq}). Prey biomass was measured acoustically (Nautical Area Scattering Coefficient (NASC) ($m^2\ nm^{-2}$)) for both zooplankton and fish. Fish biomass was estimated using 38 kHz backscatter, and consequently, only represents swim-bladdered fish. Since zooplankton and fish NASC values are from different acoustic frequencies (710 kHz, 38kHz, respectively), the values for the different taxa cannot be directly compared with each other.

Date	Distance covered (km)	DMS_g (ppb) mean ± SD	DMS_{aq} (nM) mean ± SD	Prey biomass (m^2 nm-^{2})	
				Zooplankton mean ± SD	Fish mean ± SD
23 June 2019	71.3	8.2 ± 2.0 (n = 63)	15.2 ± 3.0 (n = 63)	143.2 ± 97.5 (n = 63)	152.4± 97.5 (n = 63)
24 June 2019	38.9	3.6 ± 2.0 (n = 43)	5.4 ± 2.9 (n = 42)	101.0 ± 72.8 (n = 43)	73.0 ± 10.4 (n = 43)
26 June 2019	11.7	3.2 ± 1.3 (n = 31)	3.8 ± 0.5 (n = 31)	3.6 ± 5.4 (n = 23)	130.8 ± 48.4 (n = 23)
27 June 2019	79.2	1.9 ± 0.7 (n = 43)	5.1 ± 4.0 (n = 43)	34.2 ± 44.5 (n = 42)	215.8 ± 153.3 (n = 42)
28 June 2019	19.1	1.1 ± 0.6 (n = 14)	4.0 ± 1.0 (n = 14)	6.9 ± 6.2 (n = 2)	251.5 ± 213.4 (n = 14)
Summary	220.2	4.5 ± 3.1 (n = 194)	8.2 ± 5.7 (n = 193)	86.1 ± 90.0 (n = 173)	153.1 ± 141.3 (n = 185)
