# Supplementary material for: Natural dimethyl sulfide gradients would lead marine predators to higher prey biomass
Source: Commun Biol. 2021 Feb 1;4:149. doi: 10.1038/s42003-021-01668-3 (PMC7851116; doi:10.1038/s42003-021-01668-3)
Supplement: Supplementary file 2 — Description of Additional Supplementary Files [file 42003_2021_1668_MOESM2_ESM.pdf]

Description of Additional Supplementary Files

File name: Supplementary Data 1

Description: CSV file containing the data used to produce Figures 1-4. The CSV file contains concurrent measurements of DMSaq, DMSg, NASC (710 kHz, zooplankton), NASC (38kHz, fish), time (UTC) and position (latitude and longitude) of the concurrent measurements.

File name: Supplementary Data 2

Description: High-resolution acoustics backscatter data (710 kHz) used in the agent based model simulation to create the spatial prey grid and produce Figure 5. The CSV file provides NASC, longitude, latitude and UTC time (in mtime matlab format).
